# One Molecule, Different Circuits? What Botulinum Toxin Can and Cannot Reveal About Craniocervical Comorbidity

**DOI:** 10.3390/toxins18090358

**Published:** 2026-08-23

**Authors:** Andrea Felice Armenti, Giovanni Salti

**Affiliations:** 1 LA VISION Training Institute, Via Magenta, 5, 00185 Rome, Italy; 2 Medlight Institute, Via Monteverdi, 2, 50144 Florence, Italy; giovannisalti@gmail.com

**Keywords:** botulinum neurotoxin type A, chronic migraine, tension-type headache, temporomandibular disorders, bruxism, somatosensory tinnitus, trigeminal system, mechanistic inference

## Abstract

Botulinum neurotoxin type A (BoNT-A) is used across coexisting craniocervical conditions, and therapeutic response is often overinterpreted as evidence of a shared generator. The bruxism literature shows why. Across six controlled studies with event-level outcome measurement, reported effects on event frequency track not dose or injection field but whether the rule used to detect an event could follow the amplitude the toxin had just reduced; none yet combines a placebo arm with an amplitude-independent event definition. This targeted critical narrative review interprets representative evidence mechanistically rather than assessing efficacy. It examines chronic migraine, tension-type headache, myogenous temporomandibular disorders, and bruxism, with somatosensory tinnitus as a cross-modal boundary case. Efficacy is protocol-specific in chronic migraine and uncertain elsewhere. Tracing studies locate somatosensory routes to the cochlear nucleus in the spinal trigeminal and dorsal column nuclei; a direct mesencephalic-trigeminal-to-cochlear projection has not been demonstrated in the tracing literature reviewed, so somatic–auditory plausibility does not establish the masticatory proprioceptive route invoked by muscle-targeted rationales. Because BoNT-A affects motor output, peripheral nociceptive signaling, and muscle spindle input—the last of these probably differing in availability across injection fields—we frame it as a site-dependent, multi-output perturbation. That pharmacology is established; what is offered here is the inferential framing and the anatomical constraint following from it.

## 1. Introduction

Chronic migraine, tension-type headache (TTH), myogenous temporomandibular disorders (TMDs), and bruxism can enter the same clinical history, but their pairwise associations are not uniform. The migraine–TMD association is the best documented [1]. Associations involving bruxism depend more strongly on phenotype and ascertainment: awake bruxism has been associated with TTH, whereas evidence linking sleep bruxism to TTH or migraine is inconsistent [2]. Somatosensory tinnitus is considered separately as a cross-modal boundary case. TMD is associated with tinnitus, although this association does not establish causal direction or identify a common generator [3]. Among the pain phenotypes, anatomical proximity and partial convergence of trigeminal and upper-cervical nociceptive inputs make shared peripheral amplification an attractive hypothesis [4,5,6]. Convergence has also been proposed as a unifying network account of craniocervical comorbidity [7]. Anatomical convergence does not, however, establish that convergent inputs become functionally equivalent at the second-order neuron, and experimental characterization indicates that convergence is structured rather than indiscriminate [5]. Bruxism enters the comparison primarily as a motor behavior, whereas somatosensory tinnitus represents a distinct somatic–auditory interaction; neither should be assumed to converge with the pain phenotypes within a single pathway. The broader clinical overlap nevertheless invites an inverse inference: if one intervention improves several of these conditions, the conditions must share a generator. It is rarely asserted in that bare form, but it recurs in three patterns: a convergent anatomical substrate presented as a unifying network account, with functional equivalence at the second-order neuron assumed rather than shown [7]; a muscle-targeted rationale built on a projection the same authors call uninvestigated and speculative [8]; and a therapeutic signal read as evidence about the generating process, as when a fall in a bruxism index derived from the injected muscle’s own EMG is interpreted as fewer events [9]. Each is reasonable as a hypothesis and becomes an error only when treated as a finding.

Botulinum neurotoxin type A (BoNT-A) is an unusually informative test of that inference. It is delivered locally, has demonstrable motor target engagement, and can also alter peripheral sensory signaling. Systemically administered preventives with a defined molecular target are not delivered locally: they cannot be assigned to an anatomical field, so they cannot be used to ask whether a response depends on where the drug was placed. Yet its effects differ by injection field and by the outcome measured. OnabotulinumtoxinA is an established preventive treatment for chronic migraine, whereas use in TTH, myogenous TMD, bruxism, and somatosensory tinnitus is off-label or experimental. Even within one phenotype, pain, muscle force, event frequency, and disability need not change together.

This review asks a narrow question: what can these response patterns legitimately reveal about craniocervical comorbidity? Its thesis is that BoNT-A is not evidence for a unitary craniocervical disorder. It is better understood as a site-dependent perturbation: the same pharmacological principle acts on different exposed terminals and motor units, and the resulting dissociations can help distinguish a symptom generator from a peripheral amplifier or motor effector. Such dissociations may support differential functional engagement but cannot anatomically isolate the circuits involved. The useful scientific observation is therefore not response alone, but which outcome changes, at which site, and in what temporal order.

## 2. Scope and Inferential Framework

This targeted critical narrative review is not systematic. PubMed/MEDLINE and Europe PMC were searched through 29 July 2026 using combinations of intervention, phenotype, mechanism, and safety terms (Tables S1 and S2); targeted PubMed anatomy searches and targeted searches of ClinicalTrials.gov and the EU Clinical Trials Information System were completed on 30 July 2026 (Table S3); and two further targeted PubMed searches, on intraneuronal toxin trafficking and on facial-muscle proprioception, were completed on 2 August 2026 (Table S4), because neither topic satisfies the intervention-by-phenotype conjunction on which the principal searches were built. Reference lists were searched iteratively. A supplementary search of CENTRAL was completed on 14 August 2026 to test the completeness of trial retrieval (Section S2). It retrieved six controlled studies in bruxers that the principal searches had not identified—five of the instrumented studies analyzed in Section 4, and a placebo-controlled pilot in myofascial pain—and confirmed that the tinnitus evidence base is as sparse as described below. Embase was not searched directly, although CENTRAL indexed 256 Embase-sourced records here; with abstract-only reports excluded by design, that gap is narrowed rather than closed, and the two principal databases overlap substantially. Registry searches were confined to the tinnitus boundary case, the only phenotype whose argument turns on whether a controlled trial exists at all. Abstract-only reports were not used as evidentiary support, and no pooled analysis or formal risk-of-bias assessment was performed. For clinical questions, randomized placebo-controlled trials, syntheses that appraised study quality, and objective target-engagement measures were prioritized; for mechanistic and anatomical questions, tract-tracing studies, human histological studies, and preclinical experiments containing an internal manipulation of the proposed mechanism were prioritized instead. Selection was purposive and was not designed to support exhaustive retrieval or systematic-review claims.

Because purposive selection can favor evidence that fits a proposed framework, discordant evidence was handled by explicit rules, set out in Section S3 of the Supplementary Materials; evidence unfavorable to the framework proposed here is reported in the same terms as evidence supporting it. The review is accordingly a mechanistic interpretation of representative evidence, not a comprehensive assessment of efficacy: it neither ranks interventions nor estimates effect sizes.

The four core phenotypes were selected purposively. Chronic migraine provides an established protocol-specific indication; TTH provides a clinically adjacent but uncertain headache comparator; and myogenous TMD and bruxism provide pain and motor phenotypes exposed through masticatory injection fields. Somatosensory tinnitus is included only as an exploratory cross-modal boundary case linked to trigeminal input. Bruxism is treated as two phenotypes rather than one. Sleep bruxism carries the instrumented evidence and is examined in detail; awake bruxism, the phenotype more consistently associated with tension-type headache, is represented by a single randomized trial and is discussed only where it bears on the measurement argument. These conditions are not assumed to have equivalent epidemiology, biology, treatment evidence, or inferential weight.

**Figure 1:**
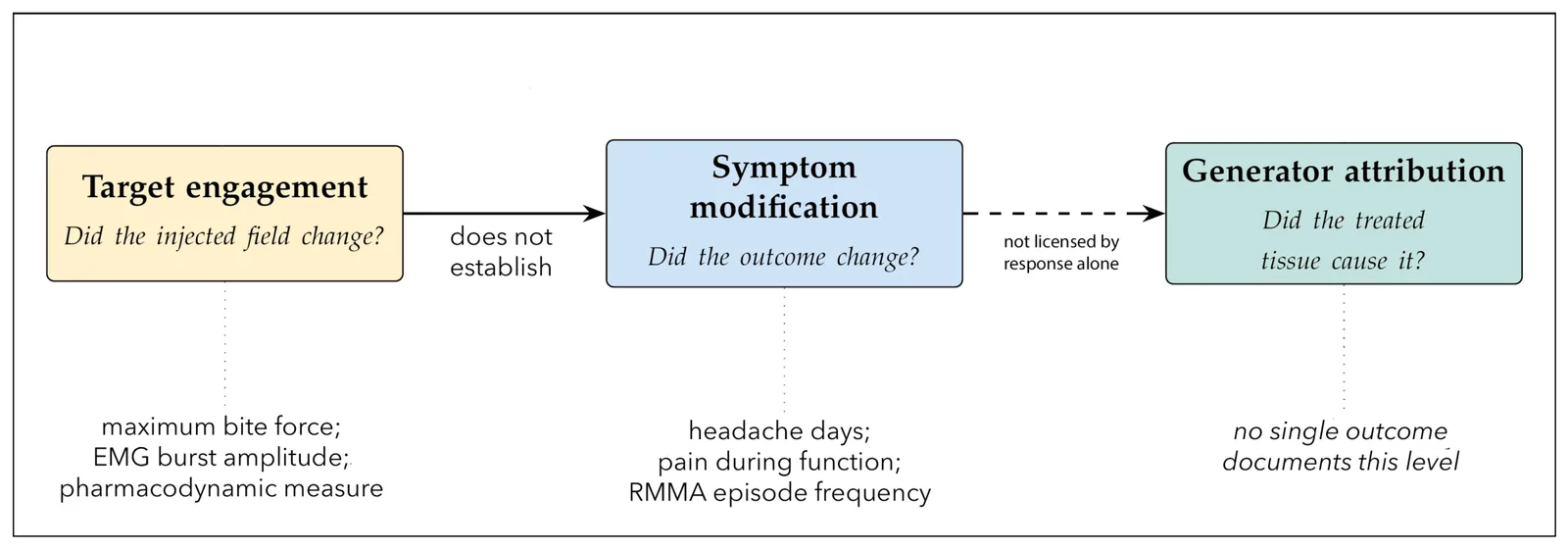
Three inferential levels and the measures that document the first two; no single outcome documents the third. Demonstrating a change at one level does not establish the next. The dashed arrow marks the inference this review argues against: therapeutic response treated as evidence of a shared generator.

Three inferential levels are kept separate. First, target engagement asks whether the injected field changed, for example as reduced bite force or electromyographic (EMG) amplitude. Second, symptom modification asks whether pain, headache days, disability, or tinnitus changed. Third, generator attribution claims that the treated tissue or pathway caused the disorder. Demonstrating target engagement confirms a local biological effect and can strengthen the mechanistic interpretation of a controlled clinical difference; it does not by itself establish that the intervention caused symptom improvement. Neither target engagement nor symptom modification alone establishes generator attribution (Figure 1).

For brevity, “BoNT-A” below denotes the serotype-A therapeutic principle. Marketed preparations and potency units are not interchangeable, and clinically observed spread depends on dose, concentration, injection volume, and technique [10], including needle depth and the number of injection sites, which together with the field determine which terminal populations are exposed. Cross-condition comparisons are therefore mechanistically suggestive, not equivalent to a controlled experiment.

## 3. One Toxin, More than One Output

BoNT-A enters susceptible nerve terminals and its light chain cleaves synaptosomal-associated protein of 25 kDa (SNAP-25), disrupting vesicle fusion mediated by soluble N-ethylmaleimide-sensitive factor attachment protein receptors (SNAREs) [10]. At the neuromuscular junction, this inhibits acetylcholine release, producing dose- and site-dependent weakness. This is the clearest target-engagement signal in masticatory applications.

The same molecular lesion is not confined to motor terminals. Preclinical and translational evidence indicates reduced stimulated release of calcitonin gene-related peptide (CGRP), substance P, and glutamate, together with altered trafficking of nociceptive receptors in peripheral sensory neurons [11,12]. A peripheral sensory action can reduce afferent drive and thereby secondarily reduce central sensitization.

That sequence does not require trans-synaptic toxin transport. Catalytically active BoNT-A does undergo retrograde axonal transport and transcytosis in animals [13,14], and this transport is causally necessary in rodents for part of the antinociceptive effect [15,16]. Three constraints separate this from human craniocervical practice: cleaved SNAP-25 marks prior catalytic activity rather than toxin presence and may itself be redistributed [17]; presence does not establish function, and at low, physiologically relevant doses catalytic activity remains confined to the motor neurons innervating the injected muscle [18,19]; and no human histological evidence exists, while syntheses of the human literature conclude that indirect effects mediated by altered sensory afference predominate [20,21].

The practical conclusion is narrow, and the rest of this review depends on it: human clinical effects of BoNT-A should not be assumed to require central toxin transport, and altered peripheral afferent input remains a sufficient candidate explanation for most observed central effects. Transport is therefore treated below as established in animals and of unproven functional relevance at the doses and fields discussed—neither assumed nor disproven. Supporting detail is given in Section S4 of the Supplementary Materials.

A third output is frequently omitted. BoNT-A also acts at gamma-motoneuron terminals innervating intrafusal fibers. In rat masseter, injection reduced spindle afferent discharge recorded in the mesencephalic trigeminal nucleus while recorded jaw-elevator tension was unchanged, although the anesthetized preparation had low baseline muscle tone [22]; in rat limb muscle, intrafusal fibers showed progressive atrophy and fusimotor denervation in parallel with extrafusal changes [23].

This third output is probably not uniformly available across injection fields, because the fields differ in receptor complement and not only in location. Masticatory muscles are richly supplied with muscle spindles, whose afferents have somata predominantly in the mesencephalic trigeminal nucleus [24,25]; masseter tissue is additionally innervated by non-spindle primary afferents, and the mandibular periodontium by a further afferent population, both with cell bodies in the trigeminal ganglion [26]. Muscles supplied by the facial nerve are generally described as lacking a proper proprioceptive system [27]; where human facial muscles have been examined histologically, typical muscle spindles were not found; their place was taken by morphologically heterogeneous sensory nerve formations, and facial proprioceptive impulses reach the central nervous system through trigeminal rather than facial branches [28,29,30]. The point is contested: spindles have been reported in mammalian facial musculature, although in that series human facial muscles contained relatively few spindles compared with other species [31].

Two qualifications, set out in Section S10, narrow this comparison: the histological studies did not examine the muscles targeted in cranial maps, and the PREEMPT map also includes temporalis, cervical paraspinal, and trapezius sites. The defensible statement is therefore not a categorical asymmetry but a difference in uniformity—a masticatory map exposes a homogeneously spindle-rich substrate, a cranial map a mixed one. It is probable rather than demonstrated, and is used only negatively: enough to block the assumption that the two fields engage identical substrates, not enough to support any positive claim about a spindle-mediated contribution in either.

Motor block, nociceptive terminal modulation, and reduced proprioceptive afferent drive are therefore three partly separable consequences of the same molecular lesion. They need not covary across injection fields, and any inference attributing a clinical effect to one of them requires that the other two be measured or excluded. They are not, however, equally well evidenced, and treating them as parallel established outputs would itself be an inferential error. Table 1 states the evidentiary tier of the two outputs that carry clinical weight. The third, reduced fusimotor and spindle afferent drive, is predominantly preclinical—single-species recordings in rat masseter and intrafusal atrophy in rat limb muscle, with no human demonstration—and intraneuronal transport is preclinical with unproven relevance at clinical doses; neither is assumed anywhere in what follows.

BoNT-A is consequently neither a pure motor block nor a pure sensory probe: injection topology determines which mixture of effects is engaged. This is a limitation for causal attribution and, equally, the basis of its experimental value, because motor and sensory outcomes can be measured separately.

## 4. The Cross-Phenotype Evidence

### 4.1. Chronic Migraine

Chronic migraine provides the strongest efficacy anchor, but the evidence is specific rather than uniform. PREEMPT 1 did not meet its prespecified primary endpoint of headache episodes, although headache-day and migraine-day secondary outcomes favored onabotulinumtoxinA; PREEMPT 2 met its headache-day primary endpoint [32,33]. Subsequent evidence syntheses support onabotulinumtoxinA over placebo, with a modest average benefit, while not showing superiority to every active preventive [34,35]. The validated paradigm uses 155–195 units across 31–39 cranial and cervical sites every 12 weeks; guidelines recommend assessing more than one cycle before classifying non-response [36,37]. These data support efficacy for a defined phenotype, preparation, and protocol, not a class effect for any headache or injection map.

Muscle relaxation alone is an inadequate account of migraine prevention. The PREEMPT field is heterogeneous in this respect: it includes muscles supplied by the facial nerve, in which an equivalent spindle complement is unlikely, alongside temporalis, cervical paraspinal, and trapezius sites, in which spindles are present (Section 3). Accounts invoking reduced proprioceptive drive are therefore less uniformly available across this field than across a masticatory one, rather than unavailable. Sensory terminal effects on CGRP release and nociceptor trafficking provide a plausible peripheral mechanism [12]. However, PREEMPT does not experimentally isolate motor from sensory action, and clinical response does not identify the original migraine generator. Its efficacy is consistent with modification of peripheral input to a chronic-migraine network; it does not establish the direction or completeness of that mechanism.

### 4.2. Tension-Type Headache

The contrast with TTH is informative. Trials differ in diagnosis, pericranial tenderness, toxin preparation, dose, injection sites, and endpoints. A systematic review of eleven reports found that ten described reduced headache intensity, but the included studies were predominantly uncontrolled cohorts with samples of 5 to 125 participants, the evidence was graded as moderate after downgrading for study design, heterogeneity, and indirectness, and the single report finding no advantage over control was randomized and placebo-controlled [38]; a later review concluded that evidence remains insufficient and noted diagnostic overlap with chronic migraine [39]. There is no validated TTH analog of PREEMPT.

Experimental work supports the possibility that sustained pericranial muscle activation can trigger headache in susceptible participants, but the relevant groups were defined by the subsequent outcome; reviews therefore treat muscle nociception as a contributor or amplifier rather than a sufficient explanation of TTH [40,41].

A favorable result after injection into tender pericranial muscles could reflect reduced peripheral nociceptive input in a selected phenotype, but cannot establish that muscle overactivity generates TTH generally; negative or inconsistent results likewise do not refute a peripheral contribution; they show only that diagnosis and injection topology have not selected a reproducible target.

### 4.3. Myogenous Temporomandibular Disorders

Here, myogenous TMD refers to masticatory muscle pain confirmed by an examiner using validated diagnostic criteria, rather than to any mixed TMD sample [42]. The TMD literature nevertheless combines different diagnostic strata and illustrates how analytic choices alter the apparent conclusion. One 2024 meta-analysis found no clear superiority over placebo for pain or functional outcomes [43]; another found a pain benefit after four weeks but no improvement in maximum mouth opening and urged caution because of bias and heterogeneity [44]. An umbrella review judged the evidence for pain reduction versus placebo to be of moderate certainty but found no superiority over standard care, and recommended BoNT-A only as a last alternative because of adverse-event concerns [45]. A recent multicenter pilot trial randomized 45 participants with jaw myalgia to either 100 units of onabotulinumtoxinA across masseter and temporalis sites or saline, and found no significant advantage in days with functional jaw pain at two months [46]. A mechanistic review similarly characterized clinical effects as modest and variable [47]. An earlier placebo-controlled pilot in 20 bruxers with myofascial pain, using 100 units across masseter and anterior temporalis, illustrates how fragile these estimates are: after correction for multiplicity, only pain during chewing and the patient’s own rating of treatment efficacy separated from placebo, and only at six months, with the active group starting from a higher baseline pain score [48]. This is active disagreement, not merely weak evidence: syntheses drawing on overlapping primary literature reach opposite judgments on whether a pain benefit versus placebo is established. Diagnostic criteria and case mix, preparation and dose, injection field and technique, comparator, primary outcome and its interval, and risk of bias can each move a pooled estimate. The safest reading is that the primary trials are not yet standardized enough for pooling to be decisive.

These findings support, at most, an adjunctive role in selected refractory myogenous pain. Reduced pain with reduced masseter force may indicate that the muscle is a modifiable amplifier. It does not distinguish nociceptor inhibition from unloading, nor prove that muscle hyperactivity was the primary disorder.

### 4.4. Bruxism

Bruxism provides the clearest within-study illustration of why target engagement and event frequency must be measured and interpreted separately—and, on inspection, of why this literature is stratified by how events are detected rather than by what the toxin does. Updated international consensus treats sleep and awake bruxism as masticatory muscle activities whose clinical relevance depends on their context, consequences, and interaction with other conditions, rather than automatically as disorders [49,50]. Assessment should distinguish subject-based, clinically based, and instrumentally based information. The Standardised Tool for the Assessment of Bruxism (STAB) provides a multidimensional framework covering bruxism status, consequences, risk factors, and comorbid conditions [51].

Six controlled studies have counted masticatory events after BoNT-A, five instrumentally and one by ecological momentary assessment. Their results align not with dose, preparation, or injection field, but with whether the rule used to detect an event could follow the amplitude the toxin had just reduced.

Where detection is anchored to an amplitude threshold fixed before injection, event counts fall. In a double-blind trial in 12 participants, ambulatory home electromyography scored events above 20% of a maximum voluntary contraction measured at baseline; masseter events fell from 2.77 to 0.15 per hour by four weeks while the uninjected temporalis was unchanged, and lowering the threshold to 10% did not alter the conclusion [52].

A placebo-controlled cross-over trial reported the opposite: the bruxism index was lower on active treatment at four weeks (mean difference −1.66, *p* = 0.003), an effect no longer present at twelve weeks [53]. It is the strongest design in the group, but it does not report whether its amplitude threshold was re-anchored, and the features usually taken to discriminate a drug effect from a detection artifact—dose-dependence, baseline-dependence, a four-week peak—do not discriminate here, because each tracks amplitude reduction. The analysis is in Section S9.

Where detection is re-anchored at each recording, or does not depend on amplitude at all, event counts do not fall. In a placebo-controlled polysomnographic trial with 200 units across masseter and temporalis, the event threshold was recalibrated to 20% of a maximum clench measured at each recording; bruxism events changed from 9.18 to 6.95 per hour on active treatment against 4.63 to 10.65 per hour on placebo (*p* = 0.09), with night-to-night variation in the placebo group spanning 122 more to 72 fewer events per night [54]. In a video-polysomnographic study of 20 participants, phasic and mixed episodes—81% of episodes at baseline—were scored from observable jaw movements on audio-video and only then confirmed against the electromyographic trace, explicitly regardless of amplitude criteria; the rhythmic masticatory muscle activity (RMMA) index did not change in either group (masseter only, 3.27 to 2.82; masseter plus temporalis, 2.81 to 2.88) while peak burst amplitude during those episodes fell by 62 to 69% in the injected muscles [55]. The remaining isolated tonic episodes were still scored against a threshold set on the baseline night, so amplitude independence applies to most of the episodes counted rather than to all of them. A later randomized, placebo-controlled polysomnographic study using 25 units per masseter reproduced the same pattern: peak amplitude fell for 12 weeks, while RMMA episodes per hour showed no treatment-related reduction [56]. The two polysomnographic studies share their first and senior authors and are not fully independent replications.

The awake phenotype adds a further route and the same result. A randomized trial in adults with temporomandibular disorders, 60 randomized and 40 analyzed, compared 40 units of incobotulinumtoxinA with electromyographic biofeedback, using smartphone-based ecological momentary assessment—amplitude-independent by construction—as the primary outcome; no behavior changed in the toxin arm to six months [57]. There was no placebo arm, and target engagement was not documented, so the null cannot be assigned to the drug rather than to the outcome.

Taken together, these six studies show neither that BoNT-A leaves event generation intact nor that it suppresses it: reported effects track whether the detection rule could follow the amplitude the toxin reduced, and the authors of the video-polysomnographic study give exactly this explanation for the discrepancy with the ambulatory data. What is missing is a single, nameable experiment—a placebo-controlled study with an event definition independent of the amplitude of an injected muscle. Until it is done, whether BoNT-A modifies the generation of masticatory events, as opposed to their measurable expression, remains open in the strict sense.

A later uncontrolled pre–post pilot in 10 participants reported a reduction in the Bruxoff bruxism index 40 days after bilateral masseter incobotulinumtoxinA [9]. Because the index was derived from the injected masseter EMG signal and the study had neither a placebo group nor an independent event marker, that decrease cannot distinguish fewer centrally generated events from attenuation of the signal used to count them.

Broader syntheses suggest possible reductions in pain, bite force, or reported events [58], but all reviews included in a recent overview had low or critically low methodological confidence [59]. A meta-epidemiological analysis also found frequent spin in bruxism trials, particularly in their conclusions [60]. Evidence that BoNT-A normalizes the process generating bruxism therefore remains weak.

### 4.5. Cross-Modal Boundary Case: Somatosensory Tinnitus

Somatosensory tinnitus is included as a cross-modal boundary case for mechanistic inference, not as a coequal indication. It is a defined phenotype, not a synonym for tinnitus coexisting with jaw symptoms: consensus criteria treat modulation by movement, temporal coupling with jaw or neck complaints, and accompanying somatic findings as convergent clues, with no single maneuver diagnostic [61]. The distinction matters because evidence from an unselected tinnitus population cannot establish an effect on tinnitus specifically linked to somatic input. The phenotype is also less discriminative than its prevalence suggests. Somatic modulation is reported in a majority of patients when broad maneuver repertoires are used, but arises in only about one in six individual maneuver trials, and in most instances the reported change is one point on a ten-point scale [62]. In a prospective cohort, the presence of somatic modulation did not influence treatment outcome [63]. Somatic modulability therefore identifies a responsive input rather than a symptom generator, and provides weak grounds for anatomical target selection.

Direct BoNT-A evidence is sparse and does not test a masseter-targeted somatosensory phenotype. In a small randomized double-blind crossover study, 30 patients received 20–50 units of BoNT-A or saline unilaterally, subcutaneously and periauricularly rather than into the masseter; 26 completed both periods [64]. Seven reported improvement after BoNT-A against two after placebo; most other comparisons were not significant, and participants were not prospectively selected for somatosensory or masseter-related tinnitus.

In a later observational chronic-migraine cohort, tinnitus was identified in only five of 57 participants, partly through retrospective questioning after an initial serendipitous response [65]. All five were migraine responders, none had bruxism, jaw pain, or TMD, and tinnitus improvement followed bilateral temporalis injections; in four patients, its duration broadly paralleled migraine improvement. These findings are hypothesis-generating but cannot distinguish a direct tinnitus effect from migraine modification, retrospective ascertainment, or correlated symptom improvement. At search close, no controlled trial of masseter-targeted BoNT-A in a prospectively defined masseter-related phenotype had been reported. Three registrations without available results were identified: a completed trial of incobotulinumtoxinA in the auricular muscles (NCT05650645) [66], a triple-blind crossover trial in tinnitus with concurrent bruxism and/or jaw-muscle myalgia (TINNITOX; CTIS 2024-518256-23-00) [67], and a registered study of botulinum toxin and facial anesthetic infiltration on tinnitus intensity in temporomandibular disorders, retrieved by the CENTRAL search [68]. As publicly registered, the TINNITOX endpoints are symptom scales; no bite-force, electromyographic, or other motor target-engagement measure is listed, so the trial cannot by itself distinguish symptom modification from masticatory target engagement or generator attribution. The registered primary and secondary endpoints are reproduced in Section S5 of the Supplementary Materials. The tinnitus literature therefore illustrates the boundary between anatomical plausibility, clinical signal, and causal attribution; it does not yet provide evidence of efficacy or field-specific circuit engagement.

A recent scoping review proposed masseter-targeted BoNT-A as a hypothesis-generating option in somatosensory tinnitus and outlined patient-selection criteria for future controlled studies [8]. Its authors state that the proposed link between masseter afferent input and the dorsal cochlear nucleus has not been investigated and remains speculative. That assessment is consistent with the present analysis and can be stated more specifically: the anatomical premise, and not only the clinical evidence, is currently unverified. Section 5 examines that premise directly, because it constrains inference beyond this phenotype.

The boundary case therefore yields a general principle, and it governs the four core phenotypes as well: anatomical plausibility is not equivalent to demonstration of a source-specific pathway, and demonstration of a pathway is not equivalent to evidence of treatment efficacy. Each step must be evidenced on its own terms. Tinnitus makes the three steps unusually easy to separate because the invoked route is nameable and has been mapped directly; where the invoked mechanism is peripheral amplification instead, the test is harder to specify, but the requirement is the same.

Table 2 summarizes, for each phenotype, the representative evidence, the preparation and injection field, the principal observation, and the inferential limit that follows from it.

## 5. What Reaches the Cochlear Nucleus: An Anatomical Constraint

The tinnitus boundary case raises a question that applies more generally: when a clinical inference invokes afferent input from a named muscle, which afferents actually reach the target nucleus? For the somatosensory–auditory interface, this is tractable, because the relevant projections have been mapped directly.

Any claim of source-specific central routing requires tract-level support, whichever phenotype invokes it.

Trigeminal input can modulate dorsal cochlear nucleus activity in animal preparations, providing an anatomical route for somatic–auditory interaction [69]. That route is, however, narrower than the general term suggests. Retrograde and anterograde tracing identifies the spinal trigeminal nucleus, principally pars interpolaris and caudalis, together with the dorsal column nuclei, as somatosensory sources projecting to the cochlear nucleus [70,71]; a direct trigeminal ganglion projection described in guinea pig was not detectable in mouse [72]. Proprioceptive information does reach the cochlear nucleus, but by two documented routes: from the neck and pinna through the dorsal column nuclei, and from extraocular muscle spindles whose cell bodies lie in the ophthalmic division of the trigeminal ganglion [73].

**Figure 2:**
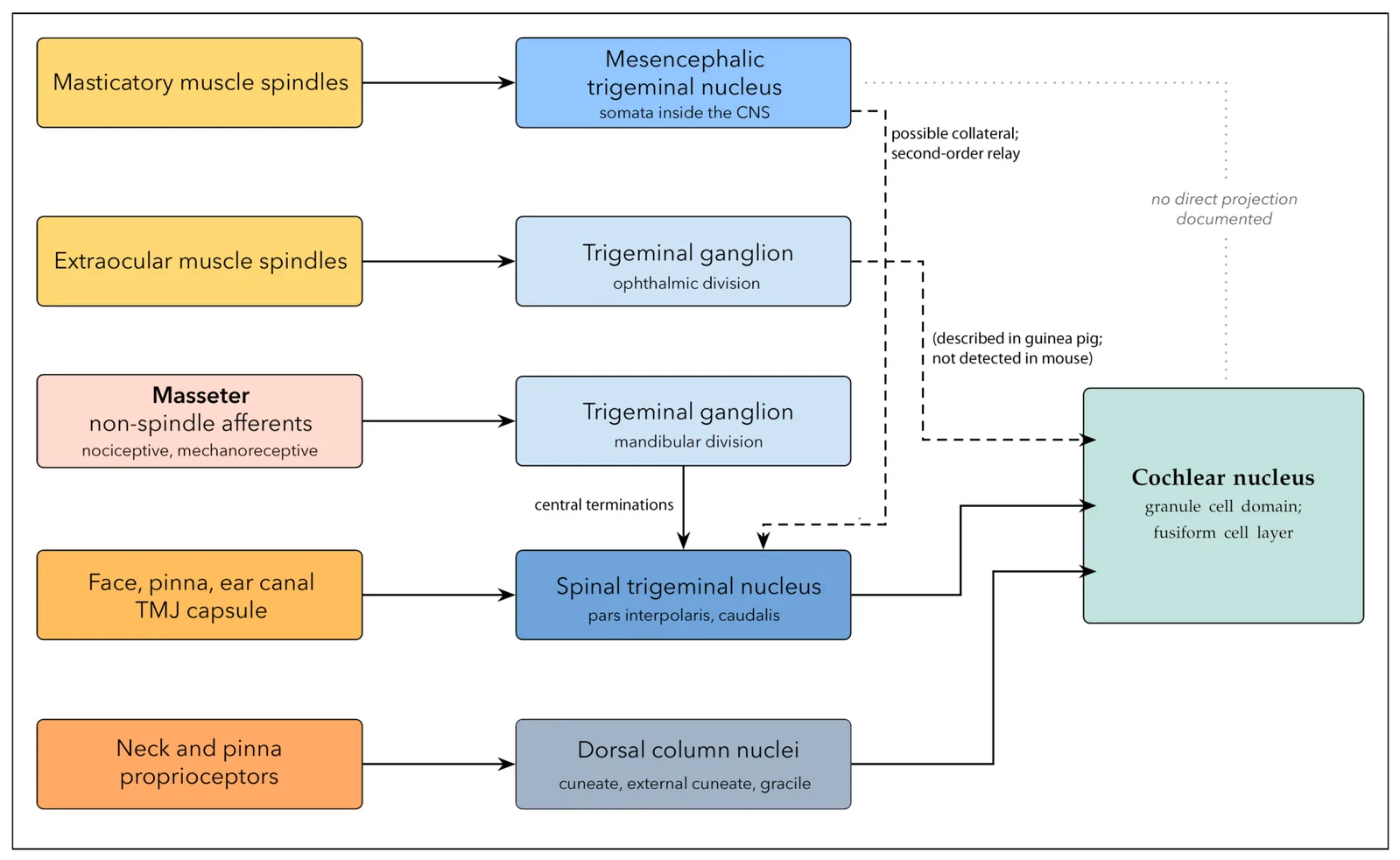
Documented somatosensory routes to the cochlear nucleus and the position of the mesencephalic trigeminal nucleus relative to them. All pathway segments derive from animal tracing studies in guinea pig, rat, mouse, cat, and macaque; none has been confirmed in humans. Masticatory afferents are separated into spindle and non-spindle populations: spindle afferents have somata predominantly in the mesencephalic nucleus, among whose enumerated efferent targets the cochlear nucleus was not identified in the literature reviewed; non-spindle masseter afferents have somata in the mandibular division of the trigeminal ganglion and terminate in the trigeminal sensory nuclear complex. Extraocular spindle afferents reach the cochlear nucleus by the ganglionic route. Solid arrows denote a pathway segment documented by tracing or anatomical mapping. Dashed arrows denote either a direct trigeminal-ganglion projection described in guinea pig but not detected in mouse, or a possible indirect mesencephalic relay involving second-order transformation, as labeled on each arrow. The dotted line denotes that no direct projection has been demonstrated in the tracing literature reviewed.

Masseter innervation is not a single afferent population. Masticatory muscle spindle afferents have somata predominantly within the mesencephalic trigeminal nucleus, whereas non-spindle primary afferents from masseter tissue also have cell bodies in the trigeminal ganglion and terminate within the trigeminal sensory nuclear complex [24,25,26]. The cochlear nucleus does not appear among the efferent targets enumerated for the mesencephalic trigeminal nucleus [25,74]. Thus, a direct mesencephalic-trigeminal-to-cochlear projection has not been demonstrated in the tracing literature reviewed. That absence cannot be generalized to all somatic input arising from masseter tissue (Figure 2).

This is an absence within a synthesis rather than a reported negative experiment, and it excludes neither an undescribed collateral nor an indirect route. A polysynaptic route through the reticular formation is possible but would not confer the source specificity a muscle-targeted rationale requires. Mesencephalic neurons also project to components of the trigeminal sensory complex, which contains the principal documented trigeminal sources projecting to the cochlear nucleus [25,70,74], so masticatory spindle input could influence that pathway indirectly—relayed and transformed at second order, though still proprioceptive in origin. The cited studies do not demonstrate completion of this source-specific chain.

Anatomical plausibility for somatic–auditory interaction is therefore not equivalent to anatomical plausibility for a masticatory proprioceptive route, and neither demonstrates that masseter pathology causes tinnitus in humans. Somatosensory plausibility supplies a mechanistic hypothesis, not evidence that BoNT-A is effective for tinnitus.

## 6. What the Pattern Reveals

Three conclusions of differing evidentiary status follow. The relevant intervention is not “BoNT-A” in the abstract but a defined preparation, dose, injection field, phenotype, and outcome set, so a PREEMPT map and a masseter map should not be pooled conceptually as a single trigeminal treatment. No adequately measured cross-phenotype dissociation is currently available, for reasons that differ by phenotype. In chronic migraine, TTH, and myogenous TMD, the outcomes required to demonstrate field selectivity have not been measured jointly. In bruxism they have, repeatedly, but this literature is stratified by detection method rather than by biology. What is missing is specific and nameable—a placebo-controlled study with an event definition independent of the amplitude of an injected muscle—and stating it that way is part of the argument rather than a gap in it. And comorbidity does not require mechanistic identity: shared anatomy may permit referral, amplification, or interaction while each phenotype retains partly independent generators and outputs.

## 7. A Staged, Falsifiable Research Program

A further uncontrolled pre–post study could identify a clinical signal but not distinguish injection-field effects from correlated symptom change or spontaneous fluctuation. What follows is therefore an illustrative program, not a protocol: three questions of increasing exposure and decreasing feasibility, rather than a single decisive trial. Stage 1 applies across the four core phenotypes; Stages 2 and 3 are confined to settings in which treatment is independently justified, or equipoise supports randomization, and TTH is excluded from them because it has no validated injection map. Each stage remains interpretable even if progression to the next is not feasible (Table 3).

Somatosensory tinnitus should not determine eligibility, allocation, stratification, sample size, or any primary or key secondary estimand. If investigated, it should be confined to an optional exploratory substudy of otherwise eligible participants meeting consensus criteria [61]. Its outcomes should remain separate from migraine and masticatory outcomes, with operational details specified in Table S7 and its accompanying notes; associations between their changes remain hypothesis-generating.

### 7.1. Stage 1: Quantifying the Perturbation Without a Drug

Provocation maneuvers are the least burdensome perturbation, but their stimulus is seldom quantified, so published rankings may index force rather than the structure generating the input: mandibular protrusion and mouth opening modulate tinnitus more often than clenching, yet the stimulus was measured in neither dataset [62,75]. Recording force, surface electromyography, applied pressure, and duration, with an uninvolved maneuver as internal control, would test whether structural differences survive adjustment. These measures remain proxies. This stage addresses measurement, not treatment.

### 7.2. Stage 2: Joint Measurement During Independently Justified Care

A prospective cohort can measure the three inferential levels jointly in patients receiving masticatory BoNT-A as clinically justified care, with the treatment decision preceding enrollment. Maximum bite force, masseter electromyographic amplitude, sensory measures, and clinical outcomes are collected at baseline, peak effect, and functional recovery, with sleep, pain, and psychosocial status as mediators and, in the TMD stratum, as diagnostic axis rather than covariate. Documented target engagement is necessary to interpret an absent clinical effect—the point on which the awake-bruxism trial above founders. Expectation, regression to the mean, and time-varying confounding remain plausible; Stage 2 tests joint measurement, not efficacy.

### 7.3. Stage 3: A Comparative Injection-Field Trial

If the earlier stages demonstrate measurable dissociation, a randomized field comparison becomes informative. In adults with chronic migraine and examiner-confirmed myogenous TMD [42], a double-blind, placebo-controlled 2 × 2 factorial design could compare PREEMPT-pattern onabotulinumtoxinA with saline and bilateral masseter-targeted onabotulinumtoxinA with saline; a four-arm factorial trial has been feasible in this comorbid population [76].

Rather than specify the trial further, the main text states the principles that make such a comparison interpretable, since these, and not the operational details, are what generalize:

- measure target engagement and clinical outcomes independently, in both fields, and infer neither from the other;
- compare injection fields prospectively within one design, because cross-literature contrasts inherit differences no later analysis removes;
- treat neither field as circuit-pure—PREEMPT includes temporalis, so the topologies overlap, and no map isolates a single afferent population;
- power the trial for its clinical outcomes, leaving interaction and field-selectivity analyses supportive unless separately powered;
- read a differential field-by-outcome profile as differential functional engagement, not as anatomical isolation of a circuit.

The two primary treatment-effect questions concern the marginal effect of PREEMPT assignment on diary-derived monthly migraine days [77] and of masseter assignment on masticatory pain during function. The instrumentally assessed sleep-bruxism subgroup is defined using current terminology and STAB domains [50,51]. Candidate estimands, outcome definitions, statistical constraints, and RMMA scoring requirements are given in Tables S6–S8 and Section S8 of the Supplementary Materials, as illustrative specifications requiring completion in a protocol and statistical analysis plan.

### 7.4. The Falsifiable Prediction

The statistical constraints on interpreting this design—the distinction between the factorial interaction and a treatment-by-outcome contrast, the powering of each, the handling of blinding guesses recorded after randomization, and the status of mediation analyses—are set out in Section S8 of the Supplementary Materials.

The falsifiable prediction is that PREEMPT assignment preferentially reduces migraine days, whereas masseter assignment preferentially reduces masticatory motor output and possibly functional pain. RMMA frequency is interpretable only if results are robust to amplitude-sensitive scoring. A differential profile would support field dependence, not discrete circuits; indistinguishable changes would weaken selectivity only if target engagement, assay sensitivity, and statistical power were adequate.

## 8. Clinical Boundaries and Safety

The framework is not a proposal to broaden off-label treatment. For TTH, TMD, bruxism, and somatosensory tinnitus, uncertainty about efficacy must be discussed explicitly, and conservative, reversible care remains the comparator. Safety is also field-dependent. In PREEMPT, adverse events were more frequent with onabotulinumtoxinA than placebo; neck pain and muscular weakness were prominent, and eyelid ptosis, myalgia, and musculoskeletal stiffness were also reported [32,33]. These events were usually mild or moderate, but they can compromise blinding and matter clinically.

Masticatory injections can produce dose- and time-dependent reductions in force and muscle volume. Reported concerns include temporary regional weakness, chewing discomfort, altered smile, dysphagia or speech effects related to spread or technique, and longer-term structural change [43,45]. Animal studies consistently raise concern about mandibular bone adaptation, while human findings are limited, mixed, and drawn from small heterogeneous samples [78,79,80]. Repeated exposure, muscle function, and bone outcomes therefore belong in long-term trials.

This constrains the staged program, and the constraint is ethical rather than methodological. Exposure is justified only where an independent therapeutic indication exists, or the evidence supports genuine equipoise; mechanistic interest is never sufficient, least of all for repeated masticatory injections. The program is ordered accordingly: Stage 1 involves no exposure, Stage 2 adds measurement without study-induced exposure, and Stage 3 is conditional on the earlier stages, on ethical and regulatory approval, and on a prespecified limit to repeated cycles. No part of this framework is a reason to inject a patient who would not otherwise be treated.

The practical advantage is diagnostic restraint. A patient may benefit because BoNT-A reduced a peripheral amplifier even when the primary disorder remains central; another may show target engagement without symptom benefit. Recording both outcomes prevents therapeutic response from being mislabeled as proof of pathophysiology.

## 9. Limitations

This review is deliberately selective and does not estimate comparative efficacy. The two principal databases overlap substantially, and Embase was not searched directly; requiring full text may have introduced availability bias. The supplementary CENTRAL search materially changed the bruxism section, which is itself evidence that the purposive strategy under-retrieved in at least one phenotype. Purposive phenotype selection, screening by a single reviewer without duplicate assessment, and the absence of formal risk-of-bias assessment further limit completeness and reproducibility. Numerical data extracted into the summary tables and into Section 4 were independently re-extracted from the source articles by the second author, blind to the first author’s extraction; discrepancies were resolved by consensus against the source. Screening and study selection remain single-reviewer. The formal apparatus of the Supplementary Material should be read accordingly: the search tables document what was done, and the design tables are illustrative candidate specifications, not a protocol and not a warrant of systematic-review rigor. Iterative reference searching and use of major evidence syntheses reduce, but do not remove, these risks. Cross-condition contrasts inherit differences in preparation, dose, injection map, eligibility, and outcome definition, so they cannot demonstrate circuit specificity by themselves. The awake-bruxism evidence rests on a single randomized trial without a placebo arm and without documented target engagement, so the phenotype most consistently associated with tension-type headache is the one the framework can least well evaluate.

The site-dependent framing presupposes that the perturbation remains substantially local. That assumption is supported at low, physiologically relevant doses [19] but is not absolute, since catalytically active toxin undergoes retrograde transport and transcytosis in animal models at doses adjacent to, rather than clearly separated from, craniocervical clinical exposures per kilogram [13,17]. Should transport prove functionally relevant at clinical doses, field comparisons would remain interpretable as comparisons of exposure topology, but not as comparisons of a locally restricted perturbation.

The anatomical argument developed for the tinnitus boundary case carries its own constraints. The tracing evidence is entirely animal, obtained in guinea pig, rat, mouse, cat, and macaque, without human confirmation, and the species differ on whether a direct trigeminal ganglion projection to the cochlear nucleus is detectable. No study identified in the targeted search injected a retrograde tracer into the cochlear nucleus and reported explicitly on mesencephalic labeling. The claim is therefore about current evidentiary status, not anatomical impossibility, and a direct tracing experiment could revise it.

The proposed program is a design hypothesis, not a completed protocol: dose selection, feasibility, subgroup power, multiplicity, and stopping rules require prospective specification, and Stage 3 in particular may prove infeasible at the sample sizes required for a powered differential field-by-outcome contrast. The framework is intended to discipline mechanistic inference, not to determine care for an individual patient.

## 10. Conclusions

The available BoNT-A evidence does not support a unitary pathophysiological account of the four core phenotypes examined in this review. Evidence for BoNT-A is strongest in protocol-defined chronic migraine, is actively contested rather than merely sparse in myogenous TMD, remains uncertain in TTH, and in bruxism shows reduced motor amplitude while reported effects on event frequency track the method used to detect an event rather than the intervention. Somatosensory tinnitus, considered separately as a cross-modal boundary case, has insufficient direct evidence to support efficacy or shared-generator claims. This uneven pattern is not a failure of the molecule. It is the observation.

When a defined preparation and injection field are paired with separate outcomes, BoNT-A can help distinguish target engagement from symptom modification and both from generator attribution, and prospectively demonstrated dissociations may identify motor effectors and peripheral amplifiers within comorbid presentations. They do not establish a common cause or map a discrete circuit. The route from masticatory spindles to the cochlear nucleus that muscle-targeted rationales invoke has not been demonstrated in the available tracing literature; that is a statement about the current state of evidence, not about anatomical possibility, and a single tracing experiment could revise it. A staged program—quantifying the provocation before perturbing it, measuring the three inferential levels jointly during care justified independently of the study, and only then comparing injection fields—would make that distinction empirically testable.

## Figures and Tables

**Table 1:** Evidentiary tier of the two outputs of the molecular lesion that carry clinical weight. The third output, reduced fusimotor and spindle afferent drive, and the separate question of retrograde axonal transport are predominantly preclinical and are described in the text; neither is assumed anywhere in this review. Only motor block is routinely measurable in humans.

Output	Evidentiary Tier	Basis	Inferential Weight
Motor block at the neuromuscular junction	Established clinical pharmacodynamic effect	Dose- and site-dependent weakness, directly measurable in humans as reduced bite force or EMG amplitude [10]	Documents target engagement in the masticatory field; makes an absent clinical effect interpretable
Modulation of peripheral nociceptive terminals	Translationally supported	Reduced stimulated release of CGRP, substance P, and glutamate, and altered nociceptor trafficking; a mechanism consistent with, but not isolated by, clinical efficacy in chronic migraine [11,12]	Supplies a plausible peripheral mechanism; not separately measurable in routine practice and not isolated by any existing trial

**Table 2:** Representative evidence, exposure, outcomes, and inferential limits across craniocervical phenotypes.

Phenotype	Representative Evidence	Preparation and Injection Field	Principal Observation	Inferential Limit
Chronic migraine	PREEMPT 1 and PREEMPT 2, randomized placebo-controlled trials; guidelines and evidence syntheses [32,33,34,36]	OnabotulinumtoxinA, 155–195 U at 31–39 cranial/cervical sites	PREEMPT 1 did not meet its primary endpoint of headache episodes; PREEMPT 2 met its headache-day endpoint	Identifies no pericranial muscular generator; does not isolate motor from sensory action
TTH	Small heterogeneous studies, predominantly uncontrolled cohorts, and systematic reviews; no validated analog of PREEMPT [38,39]	Different BoNT-A products, doses, tender-muscle criteria, and pericranial maps	Inconsistent efficacy across trials	Cannot establish TTH as a disorder of excessive muscle contraction
Myogenous TMD	Meta-analyses and umbrella review; multicenter pilot randomized trial, 45 participants with jaw myalgia [43,45,46]	Heterogeneous products and doses; usually masseter ± temporalis; pilot trial 100 U versus saline	Pain effects mixed, functional superiority inconsistent; pilot trial found no advantage at two months	Weakness and unloading may coexist with sensory effects; response does not prove muscular etiology
Bruxism	Six controlled studies with event-level outcome measurement, including four randomized placebo-controlled and two randomized with active comparators [52,53,54,55,56,57]	Four preparations at doses not interchangeable between products; masseter alone, with temporalis, or with medial pterygoid; ambulatory EMG, polysomnography, video-polysomnography, or smartphone sampling	Reported effects on event frequency track the detection rule: reductions where the amplitude threshold was fixed before injection or its anchoring unreported, none where it was re-anchored or amplitude-independent	No study combines a placebo arm with an amplitude-independent event definition; night-to-night variability is large
Somatosensory tinnitus (boundary case)	Consensus phenotyping; double-blind crossover trial, 30 enrolled and 26 completers; observational chronic-migraine cohort, 57 participants; three registrations without results [61,64,65,66,67,68]	Unilateral periauricular BoNT-A 20–50 U; cranial onabotulinumtoxinA in migraine; registered auricular and masticatory protocols	Seven versus two improved in unselected tinnitus; five of 57 migraine responders improved; no registered-trial results	No published controlled masseter-targeted result; findings cannot establish efficacy, circuit direction, or a masseter cause

**Table 3:** The three stages of the proposed program, their scope, and what each can and cannot establish.

Stage	Exposure	Phenotypes	What It Establishes, and What It Does Not
1	None beyond standardized provocation testing	All four core phenotypes	Whether modulation differs across structures once force, pressure, activity, and duration are quantified. Not a matched afferent load; does not address treatment
2	Masticatory injection delivered as clinically justified care, independently of study participation	Phenotypes in which such care is already undertaken	Whether target engagement, sensory and clinical outcomes dissociate within the same participants. Does not establish efficacy
3	Randomized injection in two fields, with placebo	Chronic migraine with myogenous TMD; sleep-bruxism subgroup	Marginal treatment effects; field-by-outcome selectivity only if separately powered. No discrete circuits, since neither field is circuit-pure

## Data Availability

No new data were created or analyzed in this study.

## Supplementary Materials

The following supporting information can be downloaded at https://www.mdpi.com/article/10.3390/toxins18090358/s1: Section S1: the search strategy, with Tables S1–S4 reporting every query as entered, its record count, and its date of execution, for PubMed/MEDLINE and Europe PMC (29 July 2026), the targeted anatomy and trial-registry searches (30 July 2026), and the targeted searches on intraneuronal toxin trafficking and facial-muscle proprioception (2 August 2026), together with notes on term normalization, selection priorities, and record flow; Section S2: the supplementary search of CENTRAL, with Table S5 reporting the queries as executed, their record counts, and the screening outcome; Section S3: the rules applied to discordant evidence; Section S4: supporting detail on retrograde and trans-synaptic transport; Section S5: the registered primary and secondary endpoints of the TINNITOX trial; Section S6: candidate estimand definitions and contrasts for Stage 3, with Table S6 giving each candidate contrast and the interpretation attached to it; Section S7: Stage 3 outcome definitions, with Table S7 giving their operational definitions and timing, including the field asymmetry in available target-engagement measures, and the specifications for the optional tinnitus substudy and for blinding assessment; Section S8: the statistical constraints on interpreting the Stage 3 factorial comparison; Section S9: why the result of the placebo-controlled cross-over trial does not discriminate a drug effect from a detection artifact; Section S10: the qualifications bounding the comparison between masticatory and cranial injection fields; and Section S11: the Stage 3 sleep-bruxism subgroup, with Table S8 setting out the requirements for rhythmic masticatory muscle activity assessment.

## Abbreviations

The following abbreviations are used in this manuscript:

BoNT-A	Botulinum neurotoxin type A
CGRP	Calcitonin gene-related peptide
CTIS	Clinical Trials Information System
EMG	Electromyography
PREEMPT	Phase III Research Evaluating Migraine Prophylaxis Therapy
RMMA	Rhythmic masticatory muscle activity
SNAP-25	Synaptosomal-associated protein of 25 kDa
SNARE	Soluble N-ethylmaleimide-sensitive factor attachment protein receptor
STAB	Standardised Tool for the Assessment of Bruxism
TMD	Temporomandibular disorder
TTH	Tension-type headache
