# Supplementary material for: One Molecule, Different Circuits? What Botulinum Toxin Can and Cannot Reveal About Craniocervical Comorbidity
Source: Toxins (Basel). 2026 Aug 23;18(9):358. doi: 10.3390/toxins18090358 (PMC13611633; doi:10.3390/toxins18090358)
Supplement: Supplementary file 1 [file toxins-18-00358-s001.zip › toxins-4508916-supplementary.pdf]

Supplementary Material

One Molecule, Different Circuits? What Botulinum Toxin Can and Cannot Reveal About Craniocervical Comorbidity

This document provides the search strategy, the rules applied to discordant evidence, supporting detail for arguments compressed in the main text, and candidate design specifications. It contains no new evidence and no claims beyond those made in the article.

S1. Search Strategy

Two bibliographic databases were searched from inception to 29 July 2026, without language restriction at the search stage. Because the phenotype searches were not designed to retrieve tract-tracing studies or unpublished trials comprehensively, two targeted PubMed anatomy searches and targeted searches of ClinicalTrials.gov and the EU Clinical Trials Information System (CTIS) were completed on 30 July 2026. Two further targeted PubMed searches, addressing intraneuronal toxin trafficking and facial-muscle proprioception, were completed on 2 August 2026; these were required because both topics fall outside the intervention-by-phenotype logic of the principal searches and would not have been retrieved by them. Reference lists of included records and of major evidence syntheses were searched iteratively. Abstract-only reports were retrieved for completeness but were not used as evidentiary support. Because the review is a targeted critical narrative synthesis rather than a systematic review, the search was designed for relevance to the stated inferential contrasts and not for exhaustive retrieval; no formal screening log or study-level risk-of-bias assessment was maintained.

Table S1. PubMed/MEDLINE searches as executed on 29 July 2026, in the order run. No filters or date limits were applied.

#	Query as entered	Records
1	`("botulinum toxins, type a"[MeSH Terms] OR "botulinum toxin"[tiab] OR "botulinum neurotoxin"[tiab] OR "onabotulinumtoxinA"[tiab] OR "abobotulinumtoxinA"[tiab] OR "incobotulinumtoxinA"[tiab] OR "prabotulinumtoxinA"[tiab] OR "BoNT-A"[tiab] OR "BoNTA"[tiab])`	25,885
2	`("migraine disorders"[MeSH Terms] OR migraine[tiab] OR "tension-type headache"[tiab] OR "tension type headache"[tiab] OR "temporomandibular joint disorders"[MeSH Terms] OR "temporomandibular disorder*"[tiab] OR "jaw myalgia"[tiab] OR "masticatory myofascial pain"[tiab] OR "myofascial pain syndromes"[MeSH Terms] OR "bruxism"[MeSH Terms] OR bruxism[tiab] OR "sleep bruxism"[tiab] OR "awake bruxism"[tiab] OR "tinnitus"[MeSH Terms] OR tinnitus[tiab] OR "somatosensory tinnitus"[tiab] OR "somatic tinnitus"[tiab])`	101,663
3	`("SNAP-25"[tiab] OR SNAP25[tiab] OR "calcitonin gene-related peptide"[MeSH Terms] OR CGRP[tiab] OR "nociception"[MeSH Terms] OR nociception[tiab] OR "muscle spindles"[MeSH Terms] OR "muscle spindle*"[tiab] OR "electromyography"[MeSH Terms] OR electromyograph*[tiab] OR "bite force"[MeSH Terms] OR "bite force"[tiab] OR "pressure pain threshold*"[tiab] OR "target engagement"[tiab] OR mechanism*[ti])`	602,489
4	`("adverse effects"[Subheading] OR "adverse effect*"[tiab] OR "adverse event*"[tiab] OR safety[tiab] OR "muscle weakness"[MeSH Terms] OR "muscle weakness"[tiab] OR "bone density"[MeSH Terms] OR "mandibular bone"[tiab] OR atrophy[tiab])`	3,941,275
5	As #2, with `"jaw myalgia"[tiab]` removed (see note below)	101,663
6	`#1 AND #5`	1,809
7	`#1 AND #5 AND #3`	385
8	`#1 AND #5 AND #4`	610

Note on search #2. PubMed flagged `"jaw myalgia"[tiab]` as a phrase not present in its phrase index. Inspection of the automatic term mapping confirmed that the phrase was omitted from the executed query, and search #5, in which the term was removed explicitly, returned an identical record count (101,663). The term therefore contributed no records, and searches #6 to #8 were built on #5. Both searches are reported here rather than only the corrected one.

Note on term normalization. PubMed mapped `"tension type headache"` and `"tension-type headache"` to the same indexed phrase, so the hyphenated form appears twice in the translated query without affecting retrieval.

Note on nested searches. Searches #7 and #8 are subsets of #6, and search #10 is a subset of #9; they narrow the

Table S2. Europe PMC searches as executed on 29 July 2026. No filters or date limits were applied.

#	Query as entered	Records
9	(TITLE_ABS:"botulinum toxin" OR TITLE_ABS:"botulinum neurotoxin" OR TITLE_ABS:"onabotulinumtoxinA" OR TITLE_ABS:"BoNT-A") AND (TITLE_ABS:"migraine" OR TITLE_ABS:"tension-type headache" OR TITLE_ABS:"temporomandibular" OR TITLE_ABS:"bruxism" OR TITLE_ABS:"tinnitus")	1,541
10	As #9, AND (TITLE_ABS:"CGRP" OR TITLE_ABS:"SNAP-25" OR TITLE_ABS:"electromyography" OR TITLE_ABS:"bite force" OR TITLE_ABS:"adverse")	472

same sets by mechanism and safety terms rather than adding records. Counts for these searches are reported for completeness and are not additive with #6 and #9.

Table S3. Targeted anatomy and trial-registry searches completed on 30 July 2026.

Source	Query as executed	Result
PubMed A1	("mesencephalic trigeminal nucleus"[tiab] OR "trigeminal ganglion"[tiab] OR "spinal trigeminal nucleus"[tiab]) AND "cochlear nucleus"[tiab]	45 records
PubMed A2	("masseter nerve"[tiab] OR "masticatory muscle afferent"[tiab] OR "masseter afferent"[tiab]) AND ("mesencephalic trigeminal nucleus"[tiab] OR "trigeminal ganglion"[tiab] OR "spinal trigeminal nucleus"[tiab])	17 records
ClinicalTrials.gov	(tinnitus AND (botulinum OR Botox OR Xeomin OR onabotulinumtoxinA OR incobotulinumtoxinA OR abobotulinumtoxinA OR prabotulinumtoxinA))	One record: NCT05650645; completed; no posted results
EU CTIS	Advanced search: Medical condition = tinnitus; no other fields or filters	One record: TINNITOX (EUCT 2024-518256-23-00); no results available

Table S4. Targeted PubMed searches on intraneuronal toxin trafficking and facial-muscle proprioception, executed on 2 August 2026. No filters or date limits were applied.

#	Query as entered	Records
A3	("botulinum toxin"[tiab] OR "botulinum neurotoxin"[tiab] OR "botulinum toxins, type a"[MeSH Terms]) AND ("axonal transport"[MeSH Terms] OR "retrograde transport"[tiab] OR "retrograde axonal transport"[tiab] OR transcytosis[tiab] OR "trans-synaptic"[tiab] OR "transsynaptic"[tiab] OR "central effect*"[tiab])	153
A4	("facial muscles"[MeSH Terms] OR "facial muscle*"[tiab] OR "mimetic muscle*"[tiab] OR "facial nerve"[MeSH Terms]) AND ("muscle spindles"[MeSH Terms] OR "muscle spindle*"[tiab] OR proprioception[MeSH Terms] OR proprioceptor*[tiab])	127

Note on searches A3 and A4. These two topics are not retrievable by the principal searches. Searches #6 to #8 require the conjunction of an intervention term and a phenotype term, and neither the literature on intraneuronal trafficking of botulinum neurotoxin nor the literature on facial-muscle proprioception satisfies both conditions; the latter satisfies neither. The same limitation applies to two sources retained from the earlier synthesis that report intrafusal and fusimotor effects in masticatory and limb muscle; these fall outside searches A3 and A4 as well, and were identified by reference chaining rather than by any documented string. Searches A3 and A4 were run to make retrieval of the trafficking and facial-proprioception literature explicit rather than incidental. Part of the trafficking literature was additionally reachable by backward reference chaining from evidence syntheses already included; the facial-muscle proprioception literature was not, and was retrieved only by search A4.

Note on term normalization in A3 and A4. PubMed translated `[tiab]` to `[Title/Abstract]` throughout and added phrase delimiters to `proprioception[MeSH Terms]` and `proprioceptor*[tiab]` in the executed query. Neither change altered the retrieval logic. The queries are reported as entered; the executed translations were

inspected and retained in the search history.

Note on targeted supplemental searches. The 30 July anatomy searches (A1, A2) were used to test the source-specific routing claim for the somatosensory–auditory interface and were supplemented by backward and forward reference chaining from the identified tracing studies. The 2 August searches (A3, A4) address two distinct claims: A3 concerns whether an intramuscular perturbation remains confined to the injected field, and A4 concerns whether the receptor complement engaged by a masticatory injection field is equivalent to that engaged by a cranial field containing muscles supplied by the facial nerve. The registry searches were intentionally restricted to the tinnitus boundary case because this was the only phenotype for which registrations were used to distinguish absence of published results from absence of an ongoing or completed trial. Eligible records were interventional registrations evaluating any botulinum-toxin preparation for tinnitus, irrespective of tinnitus phenotype, injection field, recruitment status, or results status. The returned records were screened manually for this criterion. No formal registry search was undertaken across the four core phenotypes; the registry component should therefore not be interpreted as a comprehensive trial-registry review. Registrations without results were used only to define the evidence boundary and were not treated as efficacy data.

Note on record flow. Because this is a targeted critical narrative review and not a systematic review, no screening log was maintained; no deduplicated count, screening tally, or flow diagram is therefore reported. Records were selected purposively for relevance to the inferential contrasts stated in the main text. For the clinical literature, priority was given to randomized placebo-controlled trials, evidence syntheses that appraised study quality, guidelines, consensus documents, and reports providing objective target-engagement measures. For the mechanistic and anatomical literature retrieved by searches A1 to A4, priority was given instead to tract-tracing studies, histological and immunohistochemical studies in human tissue where available, and preclinical experiments incorporating an internal manipulation of the proposed mechanism, such as blockade of axonal transport or neutralization of transported toxin. Abstract-only reports were not used as evidentiary support. Eighty sources are cited in the review, seven of which were identified by the supplementary CENTRAL search described in Section **??**.

S2. Supplementary Search of CENTRAL

The Cochrane Central Register of Controlled Trials was searched on 14 August 2026 through the Cochrane Library Search Manager. CENTRAL was at Issue 7 of 12, July 2026. The purpose was not to extend the review but to test whether the original purposive strategy had missed a controlled trial capable of changing a conclusion. No filters or date limits were applied. Queries are reported exactly as executed.

Table S5. CENTRAL searches executed on 14 August 2026. Counts are as displayed in the Search Manager; for search #3 the number of records on the Trials tab is given separately.

#	Query as entered	Records
1	`[mh "Botulinum Toxins, Type A"] OR (botulinum NEXT toxin):ti,ab,kw OR (botulinum NEXT neurotoxin):ti,ab,kw OR onabotulinumtoxinA:ti,ab,kw OR abobotulinumtoxinA:ti,ab,kw OR incobotulinumtoxinA:ti,ab,kw OR prabotulinumtoxinA:ti,ab,kw OR "BoNT-A":ti,ab,kw OR BoNTA:ti,ab,kw`	6066
2	`[mh "Migraine Disorders"] OR migraine:ti,ab,kw OR (tension NEXT type NEXT headache):ti,ab,kw OR [mh "Temporomandibular Joint Disorders"] OR temporomandibular*:ti,ab,kw OR (jaw NEXT myalgia):ti,ab,kw OR [mh "Myofascial Pain Syndromes"] OR [mh Bruxism] OR bruxism:ti,ab,kw OR [mh Tinnitus] OR tinnitus:ti,ab,kw`	19,826
3	`#1 AND #2`	586 (582 Trials)
4	`#1 AND ([mh Bruxism] OR bruxism:ti,ab,kw)`	69
5	`#1 AND ([mh Tinnitus] OR tinnitus:ti,ab,kw)`	8

Note on the structure of the search. Search #3 is the CENTRAL search proper. Searches #4 and #5 are screening subsets of #3 for the two phenotypes in which the review makes claims that a single missed trial could overturn: bruxism, where the argument concerns instrumented event counts, and somatosensory tinnitus, where the argument concerns whether controlled evidence exists at all. Records relating to chronic migraine, tension-type headache, and myogenous TMD were screened within #3 and against the evidence syntheses already cited in the main text; no separate phenotype subsets were run for them, because for those phenotypes the review relies on published syntheses rather than on the completeness of primary-trial retrieval.

Note on record provenance. Within search #3, CENTRAL reported the indexing source of each record as Embase (256), PubMed (216), ClinicalTrials.gov (95), ICTRP (39), and CINAHL (4). These counts sum to more than the number of records retrieved, because CENTRAL lists more than one indexing source for records held in several databases and none for a small number of records. Embase-sourced records are therefore partly covered by this search, although not equivalently to a direct Embase search. Records were in English (565); the seventeen non-English records, of which the largest groups were German (5), Russian (4), Spanish (4), and Chinese (2), were screened individually.

Screening rule and outcome. Screening proceeded in two stages. Records were first excluded if they were not controlled studies of a BoNT-A preparation in adults in one of the five phenotypes, if they were trial registrations or abstract-only reports, or if they were already cited in the main text or already included in one of the evidence syntheses cited there. The remaining full reports were incorporated only where they bore on an inferential contrast the review makes. For bruxism this required a reported count of masticatory events obtained by instrumented or observational recording, because the claim at issue concerns how an event is detected rather than whether symptoms improve; the controlled trials reporting only pain, bite force, muscle thickness, or comparison with splint therapy were screened and not incorporated, since the review does not estimate efficacy and those designs do not address the detection question. For the remaining phenotypes a report had to be placebo-controlled and to report a masticatory pain outcome. This is a purposive incorporation rule, not an eligibility criterion for a systematic review, and it is stated here so that a reader can see which of the retrieved records were set aside and why.

- **Bruxism (#4, 69 records).** The set comprised trial registrations, abstract-only reports, and full published studies. Six controlled studies not identified by the original strategy met the incorporation rule above and are now incorporated: an ambulatory-electromyographic randomized trial, a video-polysomnographic study, a placebo-controlled polysomnographic trial, a double-blind placebo-controlled cross-over trial with home surface electromyography, a placebo-controlled pilot in bruxers with myofascial pain, and a randomized trial in awake bruxism. Their incorporation changed the substance of the bruxism section rather than adding citations to it. Among the records screened and not incorporated were two further randomized trials in sleep bruxism, published in 2024 and 2026, whose reported outcomes were bite force,

muscle thickness, or electromyographic amplitude without any count of masticatory events; neither bears on the detection question at issue here.

- **Tinnitus (#5, 8 records).** Three records were already cited. One further registration without results was retrieved and is now cited. The remaining four concerned hemifacial spasm with auricular symptoms, post-stroke spasticity, and intratympanic injection for middle-ear myoclonic tinnitus, and are outside the phenotype and injection field considered here. The sparseness of this set is consistent with the account given in the main text.
- **Non-trial records (#3, 4 records).** Besides the 582 Trials, search #3 returned three Cochrane Reviews and one Clinical Answer. One of the reviews was the Cochrane review of botulinum toxins for migraine prevention already cited in the main text; the other two concerned interventions other than botulinum toxin.

This search was executed after the stated evidentiary cut-off of 2 August 2026 and is reported as a completeness check against that cut-off rather than as an extension of it.

S3. Handling of Discordant Evidence

Purposive selection can favor evidence consistent with a proposed framework. Because no formal risk-of-bias instrument was applied, the following rules were used instead, and are stated so that a reader can audit them against the text.

1. **Discordance is reported, not resolved.** Where two or more evidence syntheses addressing the same question reached different conclusions, both conclusions are stated in the main text. No synthesis was treated as settling a question that another synthesis contests, and no synthesis was omitted because its conclusion was inconvenient for the framework proposed.
2. **Discordance is attributed to identifiable features where possible.** Where syntheses disagreed, the main text names the features most likely to account for the disagreement: diagnostic criteria and case mix, toxin preparation, dose and dilution, injection field and technique, comparator (placebo versus standard care), the outcome treated as primary and the interval at which it was assessed, and the methodological confidence of the synthesis itself where the authors reported it.
3. **A single trial does not override a synthesis, and a synthesis does not override a single trial.** Where a recent randomized trial and an earlier synthesis pointed in different directions, both are cited and the divergence is retained as an open question rather than adjudicated.
4. **Unfavorable evidence carries the same weight as favorable evidence.** Findings that weaken the framework proposed here are reported in the same terms as findings consistent with it. These include the failure of the prespecified primary endpoint in PREEMPT 1, the negative multicenter pilot trial in jaw myalgia, the absence of a treatment-related reduction in measured RMMA frequency, the low or critically low methodological confidence of the bruxism review literature, the documented spin in bruxism trial conclusions, and the finding that somatic modulability did not predict treatment outcome in a prospective tinnitus cohort.
5. **Mechanistic and clinical literatures were appraised differently.** For clinical questions, priority was given to randomized placebo-controlled trials and to syntheses that appraised study quality. For mechanistic and anatomical questions, priority was given to studies containing an internal manipulation of the proposed mechanism (for example, blockade of axonal transport or neutralization of transported toxin) and to direct tract-tracing or histological observation. A mechanistic claim supported only by an association was not treated as equivalent to one supported by an internal manipulation.
6. **Absence of evidence is distinguished from evidence of absence throughout.** Where the review reports that something has not been demonstrated, the wording refers to the literature retrieved by the searches documented above and does not assert that the phenomenon does not exist.

This review therefore provides a mechanistic interpretation of representative evidence. It does not rank interventions, does not estimate effect sizes, and should not be cited as an efficacy assessment.

S4. Retrograde and Trans-Synaptic Transport: Supporting Detail

The main text states the practical conclusion: human clinical effects of BoNT-A should not be assumed to require central toxin transport, and altered peripheral afferent input remains a sufficient candidate explanation for most observed central effects. The supporting detail is given here so that the conclusion can be checked against the evidence rather than accepted on assertion.

What is demonstrated in animals. Catalytically active BoNT-A undergoes retrograde axonal transport and neuronal transcytosis in animal models. Cleaved SNAP-25 appears in the facial nucleus after injection into whisker-pad muscles and in retinal terminals after injection into the optic tectum, carried in non-acidified axonal compartments that partly overlap those used by tetanus neurotoxin. In rodents this transport is not merely detectable but causally necessary for part of the antinociceptive effect: intraganglionic colchicine abolishes the effect of peripherally administered BoNT-A on facial pain, and intrathecal neutralizing antitoxin abolishes the contralateral analgesic effect after unilateral hind-paw injection.

Why this does not transfer directly to human craniocervical practice. Three constraints apply. First, cleaved SNAP-25 marks prior catalytic activity rather than the presence of toxin, and cleavage fragments generated peripherally may themselves be transported; the most recent mapping study notes that hematogenous distribution followed by uptake into distant peripheral nerves would reproduce the observed bilateral pattern. Second, presence does not establish function: internalized toxin can migrate intraneuronally without detectable inhibition of distal transmission, and at low, physiologically relevant doses catalytic activity remains confined to the motor neurons innervating the injected muscle. Third, no human histological evidence exists. The strongest human observation is depression of spinal recurrent inhibition in a muscle that was not injected, which implicates cholinergic Renshaw synapses that afferent mechanisms do not readily explain; a single neurophysiological finding cannot carry the inference alone, and syntheses of the human literature conclude that indirect effects mediated by altered sensory afference predominate.

Dose. The rodent experiments cited used approximately 3–7 U/kg, a range adjacent to rather than clearly separated from the per-kilogram exposures of the craniocervical protocols considered in the main text. Interspecies potency scaling is not linear, so this adjacency neither establishes nor excludes functional relevance at clinical doses.

Consequence for the framework. Central transport is treated in the main text as established in animals, causally relevant to some rodent antinociceptive effects, and of unproven functional relevance at the doses and injection fields under discussion. Should transport prove functionally relevant at clinical doses, comparisons between injection fields would remain interpretable as comparisons of exposure topology, but their interpretation as comparisons of a locally restricted perturbation would require revision. Full citations for every statement in this section are given at the corresponding points of the main text.

S5. Registered Endpoints of the TINNITOX Trial

The public record of the EU Clinical Trials Information System registration CTIS 2024-518256-23-00 (TINNITOX), accessed 30 July 2026, specifies the following.

Primary endpoint. The difference between active treatment and placebo in change in Tinnitus Handicap Inventory score from baseline to week 18.

Secondary endpoints. Differences between active treatment and placebo in change over the same interval in headache visual analogue scale, jaw-muscle pain visual analogue scale, and EQ-5D-5L scores.

Relevance to the present review. No bite-force, electromyographic, or other motor target-engagement measure appears in the registered endpoint set. As registered, the trial may therefore inform symptomatic efficacy in tinnitus with concurrent bruxism and/or jaw-muscle myalgia, but cannot by itself separate symptom modification from masticatory target engagement, and cannot support generator attribution. This is an observation about the registered design at the date of access, not a criticism of the trial's conduct or of its suitability for its own stated objective; endpoints may be amended, and the published report should be consulted in preference to the registration.

S6. Stage 3: Candidate Estimand Definitions and Contrasts

The items below are candidate treatment-effect questions and contrasts, not complete protocol-level estimands. Before trial initiation, each estimand must specify the treatment conditions, target population, outcome variable and exact time window, strategy for each intercurrent event (including rescue medication, change in preventive treatment, repeat injection, and discontinuation), and population-level summary. Multiplicity control applies to the two primary treatment-effect questions. Secondary, mechanistic, interaction, and differential-profile analyses are reported with effect estimates and confidence intervals and are supportive or exploratory unless separately powered and included in a prospective multiplicity strategy.

Interpretation of factorial effects. The marginal effect of either assignment is an average over the other assignment. If a material PREEMPT-by-masseter interaction is present, arm-specific effects should accompany the marginal estimate and the additivity assumption should not be used to summarize the result.

Table S6. Candidate estimand definitions and contrasts for the Stage 3 comparative injection-field trial.

Level	Outcome or question	Candidate contrast and interpretation
Primary	Monthly migraine days	Effect of PREEMPT-pattern assignment versus saline, averaged over masseter assignment; the exact primary time window and intercurrent-event strategy must be fixed in the protocol
Primary	Masticatory pain during function	Effect of masseter assignment versus saline, averaged over PREEMPT-pattern assignment; the exact primary time point or window and intercurrent-event strategy must be fixed in the protocol
Secondary	Responder rate	Proportion achieving a prespecified reduction in monthly migraine days, by PREEMPT-pattern assignment
Secondary	Moderate-to-severe headache days	Effect of PREEMPT-pattern assignment
Secondary	Cross-field effect on headache	Effect of *masseter* assignment on monthly migraine days
Secondary	Cross-field effect on masticatory pain	Effect of *PREEMPT-pattern* assignment on masticatory pain during function
Factorial interaction	Non-additivity of the two assignments	PREEMPT-by-masseter interaction estimated separately within each outcome; this is not a test of field selectivity
Differential profile	Field-by-outcome selectivity	Treatment-by-outcome contrast in a joint model of prespecified standardized outcomes. This is supportive or exploratory unless separately powered with prospective multiplicity control. A structural expectation of asymmetry follows from the differing receptor complements of the two fields, but that expectation is anatomical and does not license interpreting an observed asymmetry as circuit isolation
Mechanistic	Motor target engagement, masticatory field	Effect of masseter assignment on maximum bite force and on masseter electromyographic burst amplitude
Mechanistic	Motor target engagement, PREEMPT field	Effect of PREEMPT-pattern assignment on a standardized pharmacodynamic measure in an injected PREEMPT muscle (corrugator or frontalis)
Mechanistic	Fusimotor and proprioceptive engagement, masticatory field	Effect of masseter assignment on the masseteric (jaw-jerk) reflex, normalized to maximal M-wave and to maximal voluntary electromyographic amplitude recorded at the same visit. No equivalent measure is available for the PREEMPT field; this asymmetry is a property of the anatomy under examination and is not itself evidence of differential efficacy
Mechanistic	Sensory outcome	Effect of each field assignment on pressure pain thresholds at prespecified sites
Subgroup	RMMA episode frequency	Effect of masseter assignment in the instrumentally assessed sleep-bruxism subgroup, accompanied by prespecified threshold-sensitivity analyses and non-injected corroborating signals where feasible
Exploratory	Tinnitus loudness; tinnitus-impact score	analyzed separately from all other outcomes, in the optional substudy only; hypothesis-generating and not interpreted as evidence of mediation, field selectivity, circuit direction, or a shared generator

S7. Stage 3: Outcome Definitions

Note on field asymmetry in target-engagement measures. The two injection fields cannot be characterized by an identical mechanistic panel. Masticatory muscles carry muscle spindles whose afferents have somata predominantly in the mesencephalic trigeminal nucleus, so intramuscular injection can in principle engage both extrafusal and intrafusal terminals, and the masseteric reflex provides a partially separable index of the latter. Muscles supplied by the facial nerve are generally described as lacking typical muscle spindles, and the human facial muscles examined histologically to date have been zygomaticus major, buccinator, and orbicularis oris rather than the muscles targeted in cranial injection maps; the inference to corrugator, procerus, and frontalis therefore rests on the general description of facial-nerve musculature rather than on direct observation, and the question is not closed. Two consequences follow for the trial. First, the absence of a fusimotor measure in the PREEMPT field is a limitation of the anatomy rather than of the protocol, and must not be read as absence of engagement. Second, a difference between fields in the mechanistic panel is expected by construction and cannot itself be offered as evidence of field selectivity; only the prespecified treatment-by-outcome contrast on the standardized clinical outcomes can address that question.

Optional tinnitus substudy. Tinnitus must not determine eligibility, allocation, stratification, sample size, or any primary or key secondary estimand. Otherwise eligible participants may enter an optional exploratory substudy if they meet consensus criteria for somatosensory tinnitus. Hearing-aid use, sound therapy, and other tinnitus-directed treatments should remain stable where clinically appropriate and be recorded prospectively. Somatic-modulation testing should be repeated using the same standardized maneuvers. Tinnitus outcomes should be analyzed separately from migraine and masticatory outcomes; associations between their changes are hypothesis-generating and must not be interpreted as mediation, a shared generator, or circuit-specific evidence.

Blinding assessment and analysis. Participant and assessor guesses, confidence ratings, and a prespecified blinding index are reported descriptively by randomized field and arm. Primary outcome models are not stratified or adjusted by guessed allocation because the guess is measured after randomization and may be caused by target engagement or adverse effects. Sensitivity analyses may describe the association of guesses and confidence with target engagement and outcomes, without assigning that association a causal interpretation.

Handling of infeasibility. If the sample required for the two primary marginal treatment effects cannot be reached, the program converts to the Stage 2 cohort design rather than proceeding as an underpowered factorial trial. If the primary sample is feasible but the larger sample required for a treatment-by-outcome differential contrast is not, the primary trial may proceed, but field selectivity must remain supportive or exploratory. This rule is prespecified.

Table S7. Operational definitions and timing of Stage 3 outcomes.

Outcome	Definition	Assessment
Monthly migraine days	Derived from a prospective daily headache diary using a prespecified definition, with an adequate baseline observation period consistent with international guidance for controlled trials of preventive treatment in chronic migraine	Daily diary throughout; exact primary analysis window fixed before enrollment
Masticatory pain during function	Pain intensity during standardized jaw function, recorded on a validated numerical scale in participants meeting validated criteria for myogenous TMD	Baseline and each assessment point; exact primary time point or window fixed before enrollment
Maximum bite force	Highest of three recordings per side using a calibrated gnathodynamometer at a fixed molar position, median used	Baseline and each assessment point
Masseter EMG burst amplitude	Surface electromyography at maximum voluntary contraction, electrode position marked and photographed at baseline and reproduced at each visit; median of three 5-second trials	Baseline and each assessment point
Masseteric (jaw-jerk) reflex	Surface electromyographic response of masseter to a standardized mandibular tap at a fixed jaw position and stimulus intensity, median of a prespecified number of accepted trials. Amplitude is expressed as a ratio to the maximal M-wave and to maximal voluntary electromyographic amplitude obtained at the same visit, because raw reflex amplitude is reduced by extrafusal blockade and is therefore not specific to fusimotor engagement. Interpretation rests on the time course of any divergence between the normalized ratio and the extrafusal measures, not on a single time point	Baseline, peak effect, and functional recovery
PREEMPT-field pharmacodynamic measure	Standardized surface electromyography or voluntary-contraction assessment of corrugator or frontalis; documents local motor target engagement only, and is not a surrogate for sensory engagement or antimigraine action. This measure is not equivalent in scope to its masticatory counterpart: in the masticatory field, motor engagement is accompanied by potential fusimotor engagement, whereas the muscles supplied by the facial nerve in this field are generally described as lacking a proper proprioceptive system, so no corresponding reflex measure is available	Baseline and peak effect
Pressure pain thresholds	Algometry at prespecified masticatory, pericranial, and one extracephalic control site, reported in kPa	Baseline and each assessment point
Exploratory tinnitus outcomes	One prespecified validated impact instrument (Tinnitus Functional Index or Tinnitus Handicap Inventory), repeated loudness ratings, audiometry, laterality, and repeated somatic-modulation testing; optional substudy only	Baseline and each assessment point, with the clinically meaningful change threshold fixed before enrollment
Blinding integrity	Participant and assessor guess of allocation for each field, with confidence rating	End of primary assessment period

S8. Stage 3: Statistical Constraints on Interpretation

These constraints apply to the factorial comparison described in the main text and require completion in a protocol and statistical analysis plan.

The fields are not disjoint. The PREEMPT pattern includes temporalis, so the comparison is between overlapping injection topologies rather than between separate anatomical targets. No analysis recovers a contrast between circuit-pure fields, because neither field is circuit-pure.

Interaction is not field selectivity. The PREEMPT-by-masseter interaction tests non-additivity on a single given outcome. Field selectivity is a different question and requires a treatment-by-outcome contrast across prespecified standardized outcomes. The two must not be reported interchangeably.

Powering. The trial is powered for its two primary marginal treatment-effect questions. Unless separately powered and included in a prospective multiplicity strategy, interaction and field-selectivity analyses remain supportive or exploratory, and a null interaction is not evidence of additivity. A material treatment-by-treatment interaction requires arm-specific estimates to be reported rather than marginal effects alone.

Blinding. Because muscle weakness may reveal allocation, blinding guesses should be collected and reported descriptively. They are measured after randomization and must not be used to stratify, adjust, or condition primary models.

Mediation. Analyses of sleep, pain, and psychological distress as mediators remain secondary or exploratory. They are subject to the standard identification assumptions for mediation, which a two-field factorial design does not satisfy by construction.

Interpretation of a null result. Indistinguishable changes across fields would weaken field selectivity only if documented target engagement, assay sensitivity, and statistical power were all adequate. In their absence, a null result is uninformative rather than negative.

S9. The Cross-Over Trial: Why Its Result Does Not Discriminate

The cross-over trial discussed in the main text is the strongest design among the controlled bruxism studies, and it reports a reduction in the bruxism index. Because it points against the reading offered in the main text, the analysis is set out here in full rather than compressed.

What the trial reports. Twenty-two of thirty-five participants were analyzed in a double-blind, placebo-controlled cross-over design with home surface electromyography over three consecutive nights, at doses of 60, 90, or 120 units distributed across masseter alone, masseter and temporalis, or masseter, temporalis and medial pterygoid. The bruxism index was lower on active treatment than on placebo at four weeks (mean difference −1.66, $p = 0.003$), an effect not sustained at twelve weeks ($p = 0.58$). The largest reduction occurred in the highest-dose group (mean −2.15, $p = 0.009$), and participants with a higher baseline index showed a greater change. No subjective scale differed from placebo at any time point.

What is not reported. The event definition follows established polysomnographic criteria based on an amplitude threshold. The report does not state whether that threshold was re-anchored at each recording, and no repeated maximum-clench calibration is described. This is an inference from an omission, and it is stated as such: the trial is grouped with the fixed-threshold studies because nothing in the report places it with the re-anchored ones, not because re-anchoring is excluded.

Why the usual discriminators do not discriminate. Three features of this result would ordinarily favor a genuine pharmacological effect over a measurement artifact. Each is equally predicted by the artifact account, because each tracks the magnitude and time course of amplitude reduction rather than of event generation. A larger effect at higher dose follows from greater amplitude reduction moving more events below a fixed threshold. A larger effect at higher baseline index follows from more events lying near that threshold. A peak at four weeks lost by twelve follows the pharmacodynamics of amplitude, which recover over the same interval. Dose-response, normally strong evidence of a real drug effect, therefore carries no discriminating weight in this specific comparison.

The authors' own position. The report states that the index measures the frequency and pattern of masticatory muscle activation rather than muscle strength. That is the proposition at issue in this review rather than a demonstration of it, and it is not supported by a measurement independent of amplitude.

S10. Qualifications on the Injection-Field Comparison

The main text states that a masticatory injection field and a cranial one are unlikely to expose an identical proprioceptive substrate, and uses that claim only negatively. Two qualifications bound it, and both narrow what it can support.

The histological evidence does not cover the muscles actually injected. Where human facial muscles have been examined histologically, the muscles studied were zygomaticus major, buccinator, and orbicularis oris, not the corrugator, procerus, or frontalis targeted in cranial injection maps. The inference to the upper face therefore rests on the general description of facial-nerve musculature rather than on direct observation of the relevant muscles. Direct histological examination of corrugator, procerus, and frontalis would settle the question in either direction and is feasible with existing methods; until it is done, the claim should be read as an extrapolation and not as an observation.

The cranial field is not confined to facial-nerve musculature. The PREEMPT pattern also includes temporalis, cervical paraspinal, and trapezius sites, supplied by the trigeminal, cervical, and accessory nerves, in which the argument from facial-muscle histology does not apply at all. The defensible statement is therefore not a categorical asymmetry between a facial and a masticatory field, but a difference in uniformity: a masticatory map exposes a homogeneously spindle-rich substrate, whereas a cranial map exposes a mixed one in which part of the injected volume reaches muscles that probably lack an equivalent spindle complement and part does not.

What the claim is used for. Taken together, these qualifications make the difference probable rather than demonstrated, and uncharacterized in several clinically relevant muscles. The main text uses it only to block the assumption that the two fields engage identical substrates. It is not used to support any positive claim about the magnitude or direction of a spindle-mediated contribution in either field, and nothing in the argument requires more than that.

S11. Stage 3: Sleep-Bruxism Subgroup and RMMA Scoring

The sleep-bruxism subgroup is characterized using current consensus terminology and the relevant domains of the Standardised Tool for the Assessment of Bruxism, distinguishing subject-based, clinically based, and instrumentally based information.

Table S8. Requirements for RMMA assessment in the Stage 3 subgroup.

Requirement	Specification
Recording	Repeated-night polysomnography or validated multi-night ambulatory recording; single-night recording at each time point is not adequate, given between-night variability and possible first-night effects
Scoring thresholds	Prespecified before unblinding. The primary definition is accompanied by sensitivity analyses using both absolute and within-participant relative amplitude thresholds, because no single EMG threshold can be assumed invariant to toxin-induced amplitude reduction
Amplitude dependence	Because RMMA episodes are identified from peripheral physiological signals including EMG, amplitude reduction can alter event detectability. Episode frequency is therefore not interpretable independently of amplitude unless amplitude-independent corroboration is available
Corroborating measures	Where feasible: activity in a non-injected masticatory or cervical muscle, audio/video evidence of tooth contact or orofacial movement, cortical arousal indices, and autonomic measures. Audio-video scoring of phasic and mixed episodes, confirmed against the electromyographic trace but independent of amplitude criteria, has precedent in published video-polysomnographic work and is the preferred primary route. None is treated as a fully amplitude-independent gold standard
Interpretation rule	Amplitude change without a corresponding change in episode frequency is reported as an unresolved measurement question, not as demonstrated dissociation between motor expression and event generation
